# The Role of Technology and Social Media Use in Sleep-Onset Difficulties Among Italian Adolescents: Cross-sectional Study

**DOI:** 10.2196/20319

**Published:** 2021-01-21

**Authors:** Nirosha Elsem Varghese, Eugenio Santoro, Alessandra Lugo, Juan J Madrid-Valero, Simone Ghislandi, Aleksandra Torbica, Silvano Gallus

**Affiliations:** 1 Centre for Health and Social Care Management (CERGAS) SDA Bocconi School of Management Bocconi University Milan Italy; 2 Department of Public Health Istituto di Ricerche Farmacologiche Mario Negri IRCCS Milan Italy; 3 Department of Environmental Health Sciences Istituto di Ricerche Farmacologiche Mario Negri IRCCS Milan Italy; 4 Department of Health Psychology Faculty of Health Science University of Alicante Alicante Spain

**Keywords:** sleep-onset difficulties, adolescents, social media, electronic device use

## Abstract

**Background:**

The use of technology and social media among adolescents is an increasingly prevalent phenomenon. However, there is a paucity of evidence on the relationship between frequency of use of electronic devices and social media and sleep-onset difficulties among the Italian population.

**Objective:**

The aim of this study is to investigate the association between the use of technology and social media, including Facebook and YouTube, and sleep-onset difficulties among adolescents from Lombardy, the most populous region in Italy.

**Methods:**

The relationship between use of technology and social media and sleep-onset difficulties was investigated. Data came from the 2013-2014 wave of the Health Behavior in School-aged Children survey, a school-based cross-sectional study conducted on 3172 adolescents aged 11 to 15 years in Northern Italy. Information was collected on difficulties in falling asleep over the last 6 months. We estimated the odds ratios (ORs) for sleep-onset difficulties and corresponding 95% CIs using logistic regression models after adjustment for major potential confounders.

**Results:**

The percentage of adolescents with sleep-onset difficulties was 34.3% (1081/3151) overall, 29.7% (483/1625) in boys and 39.2% (598/1526) in girls. It was 30.3% (356/1176) in 11-year-olds, 36.2% (389/1074) in 13-year-olds, and 37.3% (336/901) in 15-year-olds. Sleep-onset difficulties were more frequent among adolescents with higher use of electronic devices, for general use (OR 1.50 for highest vs lowest tertile of use; 95% CI 1.21-1.85), use for playing games (OR 1.35; 95% CI 1.11-1.64), use of online social networks (OR 1.40 for always vs never or rarely; 95% CI 1.09-1.81), and YouTube (OR 2.00; 95% CI 1.50-2.66).

**Conclusions:**

This study adds novel information about the relationship between sleep-onset difficulties and technology and social media in a representative sample of school-aged children from a geographical location that has not been included in studies of this type previously. Exposure to screen-based devices and online social media is significantly associated with adolescent sleep-onset difficulties. Interventions to create a well-coordinated parent- and school-centered strategy, thereby increasing awareness on the unfavorable effect of evolving technologies on sleep among adolescents, are needed.

## Introduction

Use of screen-based devices, such as smartphones, computer, and tablets [1], and online activities on web platforms, notably Facebook and YouTube [2,3], have grown in the recent past, particularly among adolescents. With 11 new social media users every second and each user spending more time online than before, the proportion of active social media users increased worldwide by more than 14% between January and December 2017 [4]. Specifically, among US teenagers, 95% have access to smartphones, with around 45% being online continuously [5]. The pervasive use of electronic devices has been often associated with poor sleep behaviors in adolescents. High-frequency use of social media in the hours before bed was shown to be associated with inadequate sleep [6,7]. A review of the literature found that in 90% of 67 studies conducted on the issue, the use of screen-based devices adversely affected sleep outcomes, including both sleep quantity and quality [8]. This association has been explained via three main underlying mechanisms [9], including direct time displacement from sleep duration to media use [10], increased mental, emotional, or psychological stimulation based on media content [10,11], and effects of artificial light-emitting devices on alertness, sleep health, and circadian timing [12].

More limited data are available regarding the association between social media use and sleep in the Italian population. The few studies on the issue in the United States and Northern European countries showed that the time spent on social networks, such as Facebook, as well as the time spent on the internet for playing games was significantly associated with sleep difficulties [13-15]. The share of teenagers using Facebook has decreased rapidly in the United States, with YouTube and other online social media being more commonly used [5]. The majority of studies investigating the effect of media or internet use employ measures that do not isolate the effect of online social media. Moreover, studies have rarely looked at the specific role of YouTube, the most relevant social media platform today [5]. In a recent study conducted by the Pew Research Center, 85% of the adolescents aged 13-17 years reported using YouTube, with boys reporting YouTube as their go-to platform [5]. Given the increasing prevalence of sleep problems, the continuously evolving technology, and the shift in usage preferences in social media among adolescents [16-18], the role of social media on sleep is yet to be fully elucidated.

In Italy, to our knowledge, only 2 cross-sectional studies have investigated the relationship between the use of electronic devices and sleep quality [19,20]. The first study is based on a nonrepresentative and relatively limited sample of adolescents (n=850) and finds a negative relationship between electronic device use at night and sleep quality [19]. The second and most recent study uses four waves of Health Behavior in School-aged Children (HBSC) survey data with a large sample of adolescents from 33 countries, including Italy, to show an overall negative relationship between screen time and sleep-onset difficulties [20]. The study presents an international overview of the relationship of interest, and we contribute to this literature both with additional data on use of social media and a regional focus on the issue.

Secondary data from a 2008-2009 use of time survey shows that Italians sleep less compared to the European average (8 hours, 25 minutes), and the region under study, Lombardy, scores even lower on time spent sleeping (8 hours, 18 minutes). To understand the relationship between screen time and sleep-onset difficulties among the Lombards, it should be acknowledged that the temporal profile of daily activities of Italians is different from that of the rest of the world [21]. For example, using a large (n=11,000) 2017 representative sample of the Italian adolescent population, Smorti et al (2019) show very early use of smartphones (at 11 years of age), internet dependency, and long hours spent online among Italian adolescents, with females being at higher risk of dysfunctional use [22]. Findings from a 2013-2014 dated survey of 2000 children aged 9-16 years from Denmark, Italy, the United Kingdom, Romania, and Portugal show that Italian adolescents are least likely to use internet at their own home or school and most likely to use it on the go, indicating less supervised settings [23]. This also shows that Italian adolescents are more likely to use internet on their phones. Only 7% of the Italian children own or have a mobile phone that does not connect to the mobile internet, whereas these numbers range from 23% to 41% in other countries included in the sample [23]. The report shows that Denmark is the only country that has successfully integrated internet use into daily school activities, which is also a strategic site for internet awareness and safety campaigns.

Moreover, the consequences of internet dependency per se and other lifestyle habits among Italian adolescents such as unsafe sexual habits, perception of being overweight, cyberbullying, and a range of other physical and mental health problems seem to be very severe [22]. Further comparing Italy to 41 countries for adolescents aged 11-15 years shows Italy to be among the top 2 countries to report the highest proportion of adolescents reporting multiple health problems at least once in a week, positioned among the top 11 to 20 countries that have the highest proportion of adolescents skipping breakfast and among the top 5 countries to have the highest number of 15-year-old adolescents who smoke or drink alcohol at least once a week [24].

A report compiled by the World Health Organization (WHO) shows that the prevalence of recommended levels of physical activity (at least 60 minutes per day) among adolescents (aged 11-15 years) is also lower in Italy (11%) compared to several countries including Denmark (16%, 14%, and 11% for 11-, 13-, and 15-year-olds, respectively), Estonia (16% for 11- to 15-year-olds), Latvia (19% for 11- to 15-year-olds), Poland (24% for 11- to 15-year-olds), Luxembourg (30% for ages 11-14 years) and Finland (45% for ages 10-11 years and 19% for ages 14-15 years) [25]. Using a 2010-2014 representative sample of Italian individuals, it was found that smartphone use reduces quality of face-to-face social interactions, with Lombardy displaying both an increasing smartphone penetration and the lowest fraction of people reporting to see their friends at least once a week [26].

Based on the previous considerations, our study aims to understand the relationship between frequency of use of technology (including computer, tablet, and smartphone) and social media, and sleep-onset difficulties among adolescents in a large sample from a geographical location that has not been included in studies of this type previously.

## Methods

### Sample

The HBSC study population includes school-going children aged 11, 13, and 15 years, marking the onset of adolescence. For this analysis, we used data from the HBSC wave of 2013-2014 from Lombardy. This is the most populated Italian region, including, in 2014, 10.0 million inhabitants (ie, 17% of the 60.5 million Italian people) and 0.3 million adolescents aged 11-15 years (ie, 17% of the overall population of Italian adolescents aged 11-15 years) [27]. Within the Italian HBSC, in Lombardy schools were oversampled to obtain sufficient statistical power to derive precise frequency estimates at a regional level [28]. In this specific region, a set of questions on technology and social media use was added to the standard national questionnaire to gain in-depth knowledge in this area of interest. The HBSC primarily employs cluster sampling to choose the subjects. Sampling is done in three stages, with the first being a random selection of schools from the database of all public schools in Lombardy. Within each of the 142 participating schools, a total of 235 classes were selected, with a response rate of 89.4% on average yielding a total of 210 participating classes [28]. The classes were stratified on the basis of age with the purpose of ensuring a geographical coverage that represents the actual distribution of the population of 11-, 13-, and 15-year-olds in the Lombardy region. Design weights were applied to adjust for differences in sampling frequencies, such as cases where students in certain schools were more likely to be included in the survey. Finally, all students from the selected sample of classes were invited to complete the questionnaire. Additionally, poststratification weighting of the sample was used to ensure representativeness of pupils with respect to characteristics including school denominations, school urban-rural classification, and equal representation of boys and girls. This ensures the generalizability of the results. Details on the sampling methodology of HBSC surveys have been previously described elsewhere [29]. In Lombardy, a total of 3172 adolescents aged 11, 13, and 15 years filled a self-completion questionnaire in their schools, administered in the classrooms by teachers.

The Italian study protocol was approved by the ethics committee of University of Turin. Participation in the survey was voluntary, anonymous, and with no provision of additional benefits.

Written or oral informed consent was obtained from a parent or guardian for participants younger than 16 years.

### Measures

The HBSC is an international school-based survey on adolescent health, administered every 4 years in several North American and European countries, including Italy, as a WHO collaborative cross-national study [30,31]. The survey is based on a standardized research protocol containing a theoretical framework for all the procedures, including selection of survey topics, data collection, and analysis with the objective of securing cross-national comparable data [32]. The survey instruments consist of three sets of questions: core questions similar for all participating countries to create the international dataset, optional questions on specific topics, and country-specific questions of national importance [27,31,32].

Besides general information on socioeconomic characteristics of their family, adolescents were asked to provide information on their own anthropometric variables (weight and height) and selected lifestyle habits, including tobacco smoking and alcohol drinking. The questionnaire also included specific sections on well-being, health behaviors, and social context.

#### Outcome

Difficulty to fall asleep was assessed from the question “over the last 6 months, how many times did you have difficulties in falling asleep?” The responses provided were (1) every day, (2) more than once a week, (3) about once a week, (4) once a month, and (5) rarely or never. For the analysis, we dichotomized the variable into two categories: participants with no sleep-onset difficulties (ie, adolescents with difficulties in falling asleep either once a month, rarely, or never) and participants with sleep-onset difficulties (ie, adolescents with difficulties in falling asleep either every day, more than once a week, or about once a week). This question and its cutoff have already been used in previous studies with the same sample [33]. Difficulty falling asleep is one of the four items of the HBSC symptom checklist used to measure psychological health and was shown to have high external and internal construct validity in a sample of Canadian adolescents [34]. The question about sleep-onset difficulties was also used to disentangle adolescents with severe sleep-onset difficulties (difficulty falling asleep every day) from adolescents with less severe sleep-onset difficulties (more than once a week, once a week, once a month, rarely or never).

#### Exposures

We consider four independent variables in our main analysis. Students were asked to report (1) the usage frequency of electronic devices including computers, tablets (iPad types), and smartphones for purposes such as doing homework, sending emails, chatting, tweeting, staying on Facebook, or surfing online during their free time; and (2) the use of electronic devices such as computers, consoles, tablets (iPad type), smartphones, or other devices for playing during their spare time. For both questions, the response scale provided in the questionnaire had 9 categories reported in hours per day, namely (a) never, (b) half an hour, (c) 1 hour, (d) 2 hours, (e) 3 hours, (f) 4 hours, (g) 5 hours, (h) 6 hours, and (i) 7 hours or more per day. The responses were reported for week and weekend days separately and have been shown to have considerable test-retest reliability [35,36] and acceptable criterion validity [37]. Following previous studies using the same data, we recode them to continuous measures and construct a weighted average mean, (5 × weekday + 2 × weekend day)/7 days [38]. From this weighted average, tertile categories of screen time were constructed based on the distribution of the sample. Several studies have used tertiles of screen time in similar analyses [39,40].

Students were asked to respond to how often they go on (3) social networking sites and (4) YouTube when they were connected to the internet. For both questions, the response categories provided were (1) always, (2) often, (3) sometimes, and (4) never. Likert scales of similar groupings are commonly used to indicate frequency of use of online social networking sites in several studies [41,42]. For the analysis, based on the distribution of responses, we transformed these variables into three categories, namely always, often and sometimes, or never. Information on use of social networking sites and YouTube were not collected from 11-year-olds, as social media use is restricted for children younger than 13 years in Italy as a result of Children’s Online Privacy Protection Act of 1998 (COPPA). Body mass index (BMI; calculated from self-reported weight and height in kg/m^2^) was categorized by considering the age- and sex-specific cutoff points adapted from Cole et al (2000) [43]. Validity of self-reported weight and height using the same data tested in several adolescent populations shows that there is a small underestimation of weight with very little consequence on overall results [44,45]. The students were also asked the number of times they smoked a cigarette and drank alcohol in a month. Studies using the HBSC dataset either use a dichotomous categorization of alcohol and smoking frequency [46] or retain the original variable as such [47]. However, studies also highlight the highly skewed nature of these two variables, thereby suggesting a dichotomous categorization of alcohol and smoking frequency into never and ever (at least one time in a month) [46].

### Statistical Analysis

Odds ratios (ORs) of sleep-onset difficulties (and severe sleep-onset difficulties) and corresponding 95% CIs were estimated by multiple logistic regression models, after adjustment for a number of potential confounders based on previous literature. The model was run with all confounders included simultaneously in the regression. Considered covariates included categories of age, sex, mother’s level of education (primary/secondary; high school/university; do not know), father’s level of education (primary/secondary; high school/university; do not know), BMI (underweight/normal weight; obese/overweight), tobacco smoking (never; ever) and alcohol drinking (never; ever). Further, interaction tests were also performed between use of electronic devices (and social media), and sex and age. Statistical analyses were performed in SAS (SAS Institute, Cary, NC) and Stata 15 (StataCorp, College Station, TX).

### Sensitivity Analysis

We do a range of sensitivity analyses. Previous studies indicate that screen time may differ between school days and weekends [48]. Hence, we construct a total measure of screen time (total time spent on electronic devices for general purposes and playing games) for weekdays and weekends separately and construct tertiles of total screen time from this unweighted measure of total screen time. For the second sensitivity analysis, we include school effects in the statistical models. It could be that going to certain schools might affect sleep outcomes differently for reasons including higher academic pressure or other school-related characteristics [49,50]. Finally, we test for different definitions of exposure to screen time and sleep-onset difficulties based on international health guidelines. We categorize frequency of overall use of electronic devices (total of time spent on electronic devices for general purposes and playing games) into exceeding 2 hours of daily screen exposure or not [51], and sleep-onset difficulties are defined as having difficulties in falling asleep every day or more than once a week [20,52]. Finally, family income is a potential confounder affecting the relationship between use of technology (and social media) and sleep problems [53]. However, given the unavailability of information on family income, we repeated our main analysis controlling for the perceived economic status of the family. The responses are categorized as very good, quite good, average, bad, and very bad, with 43 missing observations. Given the very few observations for the “very bad” category, we collapsed responses for bad and very bad into one category in our analysis.

## Results

Out of 3172 adolescents, 3151 (99.3%) had available information on sleep-onset difficulties. The percentage estimates of adolescents with sleep-onset difficulties were 34.3% (1081/3151) of the total sample and 30.3% (356/1176), 36.2% (389/1074), and 37.3% (336/901) for 11-year-old, 13-year-old, and 15-year-old participants, respectively (*P*=.001). Female participants presented higher levels of sleep-onset difficulties (598/1526, 39.2%) than male participants (483/1625, 29.7%; *P*<.001).

Multivariate ORs for the relationship between adolescent sleep-onset difficulties and use of selected technologies and social media are presented in Table 1.

**Table 1:** Distribution of 3172 adolescents with sleep difficulties overall and by technology and social media use, with corresponding odds ratios and 95% CIs for the full sample and sex-specific samples (Lombardy, 2014).

Technology use		Total				Males				Females		
		n^a^	Difficulty falling asleep		n		Difficulty falling asleep		n		Difficulty falling asleep	
			n (%)	OR^b^ (95% CI)			n (%)	OR (95% CI)			n (%)	OR (95% CI)
**Electronic device use for general purposes**												
	1st tertile (<0.9 hours/day)	1010	292 (28.9)	1.00^c^	557		148 (26.6)	1.00^c^	453		144 (31.8)	1.00^c^
	2nd tertile (0.9-2.1 hours/day)	1018	332 (32.6)	1.15 (0.94-1.42)	544		134 (24.6)	0.89 (0.66-1.19)	474		198 (41.8)	1.46 (1.08-1.97)
	3rd tertile (≥2.2 hours/day)	1059	437 (41.3)	1.50 (1.21-1.85)	483		189 (39.1)	1.74 (1.30-2.32)	576		248 (43.1)	1.30 (0.96-1.77)
	*P* value for trend	N/A^d^	N/A	<.001	N/A		N/A	<.001	N/A		N/A	.09
**Electronic device use for playing games**												
	1st tertile (<0.8 hours/day)	1149	376 (32.7)	1.00^c^	406		109 (26.9)	1.00^c^	743		267 (35.9)	1.00^c^
	2nd tertile (0.8-1.7 hours/day)	853	286 (33.5)	1.21 (0.98-1.49)	519		149 (28.7)	1.13 (0.83-1.55)	334		137 (41.0)	1.24 (0.93-1.67)
	3rd tertile (≥1.8 hours/day)	1093	401 (36.7)	1.35 (1.11-1.64)	665		214 (32.2)	1.25 (0.92-1.68)	428		187 (43.7)	1.46 (1.11-1.91)
	*P* value for trend	N/A	N/A	.003	N/A		N/A	.15	N/A		N/A	.007
**Use of social networking sites**												
	Never/rarely	701	224 (32.0)	1.00^c^	397		104 (26.2)	1.00^c^	304		120 (39.5)	1.00^c^
	Often	650	240 (36.9)	1.16 (0.91-1.48)	345		109 (31.6)	1.30 (0.92-1.84)	305		131 (43.0)	1.04 (0.74-1.47)
	Always	599	256 (42.7)	1.40 (1.09-1.81)	255		89 (34.9)	1.69 (1.17-2.45)	344		167 (48.6)	1.23 (0.87-1.73)
	*P* value for trend	N/A	N/A	.008	N/A		N/A	.005	N/A		N/A	.25
**Use of YouTube**												
	Never/rarely	412	113 (27.4)	1.00^c^	209		44 (21.1)	1.00^c^	203		69 (34.0)	1.00^c^
	Often	854	312 (36.5)	1.55 (1.18-2.04)	434		127 (29.3)	1.41 (0.94-2.13)	420		185 (44.1)	1.66 (1.14-2.42)
	Always	683	294 (43.1)	2.00 (1.50-2.66)	354		130 (36.7)	2.04 (1.35-3.10)	329		164 (49.8)	1.91 (1.29-2.84)
	*P* value for trend	N/A	N/A	<.001	N/A		N/A	.001	N/A		N/A	.001

^a^The sum does not add up to the total because of some missing values and exclusion of age 11 for social media measures.

^b^OR: odds ratio. All ORs were estimated using unconditional multiple logistic regression models after adjustment for age and sex of the child, mothers’ and fathers’ highest level of education, tobacco and alcohol use among adolescents, and BMI.

^c^Reference category.

^d^N/A: not applicable.

Sleep-onset difficulties were more common among adolescents with frequent use of electronic devices (OR 1.50 for ≥2.2 vs <0.9 hours/day; 95% CI 1.21-1.85; *P<.*001), use of electronic devices for playing games (OR 1.35 for ≥1.8 vs <0.8 hours/day; 95% CI 1.11-1.64; *P*=.003), online social network use (OR 1.40 for always vs never or rarely; 95% CI 1.09-1.81; *P*=.008), and YouTube use (OR 2.00 for always vs never or rarely; 95% CI 1.50-2.66; *P*<.001). Table 1 also shows the sex-specific ORs for the relationship between adolescent sleep quality and the use of selected technologies and social media. OR for sleep-onset difficulties associated with the use of YouTube was consistently large and significant in both sexes (OR 2.04 for always vs never or rarely; 95% CI 1.35-3.10; *P*=.001 for males and OR 1.91; 95% CI 1.29-2.84; *P*=.001 for females). When considering electronic device use for general purposes and use of social networking sites, the OR was significant only for males (OR 1.74 for high vs low use; 95% CI 1.30-2.32; *P*<.001 and OR 1.69; 95% CI 1.17-2.45; *P*=.005, respectively), whereas when considering use of electronic device for playing games, the OR was significant only for females (OR 1.46; 95% CI 1.08-1.97; *P*=.007).

Table 2 shows the multivariate ORs for the relationship between sleep-onset difficulties and use of selected technologies and social media stratified by age. Sleep-onset difficulties were more common among 11-year-old school-aged children with frequent use of electronic devices for general purposes (OR 1.48 for ≥2.2 vs <0.9 hours/day; 95% CI 1.03-2.13; *P*=.03) and electronic devices for playing games (OR 1.55 for ≥1.8 vs <0.8 hours/day; 95% CI 1.10-2.18; *P*=.01). Among 13-year-old adolescents, sleep-onset difficulties were more frequent among those with the use of electronic devices for general purposes (OR 1.85 for ≥2.2 vs <0.9 hours/day; 95% CI 1.29-2.66; *P*<.001) and YouTube (OR 1.88 for always vs never or rarely; 95% CI 1.26-2.81; *P*=.002). Finally, sleep-onset difficulties were more common among 15-year-old adolescents with frequent use of social networking sites (OR 1.58 for always vs never or rarely; 95% CI 1.08-1.78; *P*=.02) and use of YouTube (OR 2.10 for always vs never or rarely; 95% CI 1.39-3.18; *P*<.001).

**Table 2:** Distribution of 3172 adolescents with sleep difficulties overall and by technology and social media use, with corresponding odds ratios and 95% CIs for age-specific samples (Lombardy, 2014).

Technology use		11-year-old adolescents			13-year-old adolescents			15-year-old adolescents		
		n^a^	Difficulty falling asleep		n	Difficulty falling asleep		n	Difficulty falling asleep	
			n (%)	OR^b^ (95% CI)		n (%)	OR (95% CI)		n (%)	OR (95% CI)
**Electronic device use for general purpose**										
	1st tertile (<0.9 hours/day)	569	163 (28.7)	1.00^c^	264	71 (26.9)	1.00^c^	177	58 (32.8)	1.00^c^
	2nd tertile (0.9-2.1 hours/day)	334	95 (28.4)	1.02 (0.73-1.42)	370	129 (34.9)	1.41 (0.98-2.05)	314	108 (34.4)	0.98 (0.65-1.49)
	3rd tertile (≥2.2 hours/day)	244	89 (36.5)	1.48 (1.03-2.13)	417	184 (44.1)	1.85 (1.29-2.66)	398	164 (41.2)	1.15 (0.77-1.72)
	*P* value for trend	N/A^d^	N/A	.03	N/A	N/A	<.001	N/A	N/A	.51
**Electronic device use for playing games**										
	1st tertile (<0.8 hours/day)	468	129 (27.6)	1.00^c^	315	110 (34.9)	1.00^c^	366	137 (37.4)	1.00^c^
	2nd tertile (0.8-1.7 hours/day)	328	98 (29.9)	1.09 (0.76-1.56)	292	103 (35.3)	1.34 (0.93-1.94)	233	85 (36.5)	1.21 (0.83-1.76)
	3rd tertile (≥1.8 hours/day)	351	121 (34.5)	1.55 (1.10-2.18)	447	171 (38.3)	1.26 (0.90-1.77)	295	109 (37.0)	1.28 (0.90-1.83)
	*P* value for trend	N/A	N/A	.01	N/A	N/A	.19	N/A	N/A	.17
**Use of social networking sites (Facebook)**										
	Never/rarely	—^e^	—	—	440	145 (33.0)	1.00^c^	261	79 (30.3)	1.00^c^
	Often	—	—	—	323	121 (37.5)	1.13 (082-1.57)	327	119 (36.4)	1.22 (0.84-1.78)
	Always	—	—	—	291	120 (41.2)	1.30 (0.93-1.83)	308	136 (44.2)	1.58 (1.08-2.32)
	*P* value for trend	—	—	—	N/A	N/A	.13	N/A	N/A	.02
**Use of YouTube**										
	Never/rarely	—	—	—	199	54 (27.1)	1.00^c^	213	59 (27.7)	1.00^c^
	Often	—	—	—	451	164 (36.4)	1.55 (1.04-2.31)	403	148 (36.7)	1.50 (1.02-2.21)
	Always	—	—	—	404	168 (41.6)	1.88 (1.26-2.81)	279	126 (45.2)	2.10 (1.39-3.18)
	*P* value for trend	—	—	—	N/A	N/A	.002	N/A	N/A	<.001

^a^The sum does not add up to the total because of some missing values and exclusion of age 11 for social media measures.

^b^OR: odds ratio. All ORs were estimated using unconditional multiple logistic regression models after adjustment for age and sex of the child, mothers’ and fathers’ highest level of education, tobacco and alcohol use among adolescents, and BMI.

^c^Reference category.

^d^N/A: not applicable.

^e^Not available.

In the analysis of severe sleep-onset difficulties among adolescents, l0.3% (325/3151) of adolescents had severe sleep-onset difficulties; this percentage was 11.1% (130/1176) among 11-year-olds, 10.7% (115/1074) among 13-year-olds, and 8.9% (80/901) among 15-year-olds (*P*=.24). Severe sleep-onset difficulties were more common among adolescents with frequent use of electronic devices for general purposes (OR 1.53 for ≥2.2 vs <0.9 hours/day; 95% CI 1.10-2.13; *P*=.01), electronic devices for playing games (OR 1.73 for ≥1.8 vs <0.8 hours/day; 95% CI 1.16-2.58; *P*=.001), online social networks (OR 1.73 for always vs never or rarely; 95% CI 1.16-2.58; *P*=.007), and YouTube (OR 3.18 for always vs never or rarely; 95% CI 1.89-5.34; *P****<***.001) (Table 3).

**Table 3:** Distribution of 3172 adolescents with severe sleep difficulties overall and by technology and social media use and corresponding odds ratios and 95% CIs (Lombardy, 2014).

Technology use		n^a^	Difficulty falling asleep	
			n (%)	OR^b^ (95% CI)
**Electronic device use for general purpose**				
	1st tertile (<0.9 hours/day)	1010	88 (8.7)	1.00^c^
	2nd tertile (0.9-2.1 hours/day)	1018	94 (9.2)	1.08 (0.77-1.51)
	3rd tertile (≥2.2 hours/day)	1059	138 (13.0)	1.53 (1.10-2.13)
	*P* value for trend	N/A^d^	N/A	.01
**Electronic device use for playing games**				
	1st tertile (<0.8 hours/day)	1149	106 (9.2)	1.00^c^
	2nd tertile (0.8-1.7 hours/day)	853	77 (9.0)	1.11 (0.78-1.57)
	3rd tertile (≥1.8 hours/day)	1093	138 (12.6)	1.73 (1.27-2.35)
	*P* value for trend	N/A	N/A	.001
**Use of social networking sites (Facebook)**				
	Never/rarely	701	56 (8.0)	1.00^c^
	Often	650	56 (8.6)	1.01 (0.66-1.54)
	Always	599	83 (13.9)	1.73 (1.16-2.58)
	*P* value for trend	N/A	N/A	.007
**Use of YouTube**				
	Never/rarely	412	22 (5.3)	1.00^c^
	Often	854	68 (8.0)	1.42 (0.83-2.43)
	Always	683	105 (15.4)	3.18 (1.89-5.34)
	*P* value for trend	N/A	N/A	<.001

^a^The sum does not add up to the total because of some missing values.

^b^OR: odds ratio. All ORs were estimated using unconditional multiple logistic regression models after adjustment for age and sex of the child, mothers’ and fathers’ highest level of education, tobacco and alcohol use among adolescents, and BMI.

^c^Reference category.

^d^N/A: not applicable.

Finally, we did not find any statistically significant interaction between use of electronic devices (and social media) and sex and age (results not shown). Tables S1, S2, and S3 in Multimedia Appendix 1 report the results of three sensitivity analyses, respectively: analysis of screen exposure by weekday and weekend (Table S1), including school effects in the statistical models (Table S2) and using a different definition of screen exposure and sleep-onset difficulties (Table S3). In all cases similar results were found in comparison with the previous analyses. Our results remain robust after controlling for perceived economic status (Table S4). Moreover, respondents perceiving worse economic status also reported higher sleep-onset difficulties (results not shown).

## Discussion

### Principal Findings

Our data confirms that exposure to screen-based devices, online social networking sites, and video-sharing platforms is significantly associated with difficulties in falling asleep among adolescents. Our study adds to the literature by extending the findings to Lombardy, a geographical region that has not been included in studies of this type previously. Moreover, for the first time we show that use of YouTube, an online video-sharing platform, is related to sleep-onset difficulties among adolescents.

Our findings are in broad agreement with those from a meta-analysis of 20 cross-sectional studies (N=125,198) showing 53% higher odds of poor sleep quality among those with consistent bedtime media use [54]. Previous literature also shows that the use of computers and smartphones among adolescents was associated with daytime sleep-related behaviors such as sleepiness and fatigue [55], shorter sleep duration, later bedtime, and unfavorable changes in sleep habits over time [56]. Use of PC in particular has been shown to be associated with reduced sleep duration, poor sleep, and sleep-onset latency [1,57,58]. Smartphones are easy to carry around and easier to take to bed, making it an imperative tool for adolescents. Both overall and late-night cellphone use among adolescents in several countries has been associated with several sleep difficulties [59,60]. Use of cellphones, particularly for nighttime texting, was associated with insufficient sleep [61]. In Italy, over 72% of adolescents aged 11-17 years access the internet through smartphones, with talks on a potential “no mobile phone phobia” law being drafted, particularly aiming at the younger generation [62].

Given that adolescents use not just one device, but multiple devices at the same time, our results confirm previous findings on the relationship between multiple device use and sleep problems [1]. Our results are in line with the recent findings from the international study on use of technology and sleep-onset difficulties among adolescents using the same data and variables and extend the findings to a regional level [20]. Additionally, we also confirm an exposure-response relationship between the use of electronic devices and sleep difficulties [1,17].

Given the multifunctionality of electronic devices, such as for doing homework or social networking, it is important to have specific information on the kinds of activities carried out on these platforms. Information on frequency of use of each individual social networking site is relatively less available and more valuable [6]. Information on the kind of social media used also signals the nature of the activity, such as following vloggers on YouTube or connecting with peers on Facebook. We find that use of online social media and use of YouTube among adolescents are associated with higher odds of sleep-onset difficulties. A cross-sectional survey based on 467 Scottish adolescents (11-17 years old) found that overall social media use, particularly at night, was associated with poor sleep quality [63]. In addition to sleep-onset difficulties as an outcome, various studies use standardized measurements of sleep, including the Pittsburgh Sleep Quality Index, to find significant associations with social media use in general or specific sites such as Facebook [64,65]. Using data from Millennium Cohort Study, Kelly et al (2019) found significant associations between higher frequency of weekday social media use (reported in hours) and sleep problems including inadequate sleep, sleep latency, and sleep disruption [53]. Prior studies showed that the adverse effect of social media use on sleep is mediated by cognitive activation [66] and through a behavioral effect of fear of missing out leading to delayed sleep onset [67]. Finally, our findings suggest a dose-response effect of online social media use on sleep-onset difficulties. These findings are similar to those from a recent cross-sectional study on Canadian adolescents linking cumulative effect of social media use on reduced sleep duration [14].

The use of YouTube has been steadily growing every year since its launch in 2005, and it became the most popular platform among young adults in the United States in 2018 [5]. To our knowledge, our study was the first to investigate the association between YouTube use and sleep-onset difficulties among adolescents. YouTube is a video-intensive platform with some unique characteristics including asymmetric relationships, greater self-disclosure, higher personal revelation, and developing parasocial relationships, all contributing to supplanting real relationships [68]. YouTube addiction follows the same model as internet addiction, with the frequent use of YouTube being reinforced by one-sided virtual relationships with YouTubers [68]. We found a significant association between YouTube use and sleep-onset difficulties, overall and consistently in strata of sex and age. The same relationship was also observed when considering a stricter sleep definition (ie, severe sleep difficulties).

Potential limitations of our analysis include methodological drawbacks inherent to the cross-sectional study design. First, cross-sectional designs have no dimension of time; hence, it is impossible to conclude a causal relationship. Given that reverse causality (ie, adolescents use more electronic devices and social media because of difficulties in falling asleep) could not be ruled out, our findings should be confirmed by longitudinal data.

Second, our study uses a self-reported measure of sleep latency and screen time. Self-reported sleep measures in children need to be accurate for precise identification of sleep problems [69]. Although some studies showed an overestimation of sleep latency among both adolescents and adults [70,71], self-reported sleep latency and other sleep measures obtained some consensus with objective measurements [72,73]. Our measure of sleep latency may also suffer from recall bias given the 6-month period retrospective nature of the question. However, this question has already been used and validated in different studies [20,34,74]. These types of bias could be overcome in the future by the use of apps tracking sleep quality, which are some of the most used health apps among patients and citizens [75]. In addition, in this study only sleep-onset difficulties are analyzed, whereas various aspects of sleep including sleep duration, sleep quality, other sleep disturbances, and their differences between week and weekend days should be explored in future studies. Although frequency of use of electronic devices and other social media has been self-reported, they have been shown to have considerable reliability and validity [35,37]. Self-reported measures of screen time are an inexpensive and easier means of data collection in large samples and give us detailed information on the context of the activity.

Third, we do not have data at the national level, but only for Lombardy. Although the data is geographically limited, our study population was oversampled to obtain a representative sample of adolescents aged 11, 13, and 15 years at a regional level. Moreover, Lombardy represents the most populous and richest region in Italy, accounting for one-sixth of the country’s population and one-fifth of the gross domestic product. Finally, the selection of a region from Northern Italy, which is more digitally connected than Southern Italy, fits the goal of our study.

In addition, our sample includes only young adolescents aged 11-15 years who are enrolled in schools. Although findings cannot be generalized to older adolescents, our results are crucial given that younger adolescents are at higher risk of compulsive internet use [76]. The strengths of our study include the relatively large sample size and the availability of information to control for socioeconomic characteristics and risk behavior of adolescents, thereby strengthening the internal validity of our study and the representativeness of the sample at a regional level. Moreover, to our knowledge, this is the first study to examine the relationship between screen time, social media use, and sleep-onset difficulties in a geographical location that has not been studied before in the context of this research question. Exploiting the data on frequency of use of YouTube, a “parasocial networking site,” we also study for the first time the relationship between YouTube use and sleep-onset difficulties among adolescents.

### Conclusion

Our study shows that exposure to screen-based devices and online social media is associated with adolescent sleep-onset difficulties. These findings may have important public health implications. In fact, they support interventions and guidelines to minimize excess use of technology among adolescents. In line with the recommendations proposed by several pediatric societies, including the Italian Pediatric Society, it is advised to limit the use of tablets or other electronic devices after dinner to ensure better sleep [77]. The findings also call on social media industries to be an equal player in protecting the health and well-being of young users by considering them as potential users of their services [78]. Moreover, interventions should include contributions from health professionals and educators to create a well-coordinated parent-centered strategy to increase awareness on evolving technologies, social media platforms, and the deleterious effects on sleep among adolescents.

## Appendix

Multimedia Appendix 1 Supplementary tables.

## Abbreviations

BMI: body mass index
HBSC: Health Behavior in School-aged Children
OR: odds ratio
WHO: World Health Organization

## References

[1] Hysing M, Pallesen S, Stormark KM, Jakobsen R, Lundervold AJ, Sivertsen B (2015). Sleep and use of electronic devices in adolescence: results from a large population-based study. BMJ Open.

[2] Gradisar M, Wolfson AR, Harvey AG, Hale L, Rosenberg R, Czeisler CA (2013). The sleep and technology use of Americans: findings from the National Sleep Foundation's 2011 Sleep in America poll. J Clin Sleep Med.

[3] Uhls YT, Ellison NB, Subrahmanyam K (2017). Benefits and Costs of Social Media in Adolescence. Pediatrics.

[4] (2018). Global Digital Report.

[5] Anderson M, Jiang J (2018). Teens, social media & technology 2018.

[6] Shimoga SV, Erlyana E, Rebello V (2019). Associations of Social Media Use With Physical Activity and Sleep Adequacy Among Adolescents: Cross-Sectional Survey. J Med Internet Res.

[7] Reynolds AC, Meltzer LJ, Dorrian J, Centofanti SA, Biggs SN (2019). Impact of high-frequency email and instant messaging (E/IM) interactions during the hour before bed on self-reported sleep duration and sufficiency in female Australian children and adolescents. Sleep Health.

[8] Hale L, Guan S (2015). Screen time and sleep among school-aged children and adolescents: a systematic literature review. Sleep Med Rev.

[9] LeBourgeois MK, Hale L, Chang A, Akacem LD, Montgomery-Downs HE, Buxton OM (2017). Digital Media and Sleep in Childhood and Adolescence. Pediatrics.

[10] Cain N, Gradisar M (2010). Electronic media use and sleep in school-aged children and adolescents: A review. Sleep Med.

[11] Garrison MM, Liekweg K, Christakis DA (2011). Media use and child sleep: the impact of content, timing, and environment. Pediatrics.

[12] Chang A, Aeschbach D, Duffy JF, Czeisler CA (2015). Evening use of light-emitting eReaders negatively affects sleep, circadian timing, and next-morning alertness. Proc Natl Acad Sci U S A.

[13] Arora T, Broglia E, Thomas GN, Taheri S (2014). Associations between specific technologies and adolescent sleep quantity, sleep quality, and parasomnias. Sleep Med.

[14] Sampasa-Kanyinga H, Hamilton HA, Chaput J (2018). Use of social media is associated with short sleep duration in a dose-response manner in students aged 11 to 20 years. Acta Paediatr.

[15] Hawi NS, Samaha M, Griffiths MD (2018). Internet gaming disorder in Lebanon: Relationships with age, sleep habits, and academic achievement. J Behav Addict.

[16] Ferrie JE, Kumari M, Salo P, Singh-Manoux A, Kivimäki M (2011). Sleep epidemiology--a rapidly growing field. Int J Epidemiol.

[17] Twenge JM, Krizan Z, Hisler G (2017). Decreases in self-reported sleep duration among U.S. adolescents 2009-2015 and association with new media screen time. Sleep Med.

[18] Rowshan Ravan A, Bengtsson C, Lissner L, Lapidus L, Björkelund C (2010). Thirty-six-year secular trends in sleep duration and sleep satisfaction, and associations with mental stress and socioeconomic factors--results of the Population Study of Women in Gothenburg, Sweden. J Sleep Res.

[19] Bruni O, Sette S, Fontanesi L, Baiocco R, Laghi F, Baumgartner E (2015). Technology Use and Sleep Quality in Preadolescence and Adolescence. J Clin Sleep Med.

[20] Ghekiere A, Van Cauwenberg J, Vandendriessche A, Inchley J, Gaspar de Matos M, Borraccino A, Gobina I, Tynjälä J, Deforche B, De Clercq B (2019). Trends in sleeping difficulties among European adolescents: Are these associated with physical inactivity and excessive screen time?. Int J Public Health.

[21] Boffi M, Colleoni M, Del Greco M (2014). Night-time Hours and Activities of the Italians. Articulo-Journal of Urban Research.

[22] Smorti M, Milone A, Gonzalez Gonzalez J, Vitali Rosati G (2019). Adolescent selfie: an Italian Society of Paediatrics survey of the lifestyle of teenagers. Ital J Pediatr.

[23] Mascheroni G, Ólafsson K, Cuman A, Dinh T, Haddon L, Jørgensen H (2013). Mobile internet access and use among European children: initial findings of the Net Children Go Mobile project. http://eprints.lse.ac.uk/54244/1/Mobile%20internet%20access%20and%20use%20among%20European%20children_NCGM.pdf.

[24] (2016). Growing up unequal: gender and socioeconomic differences in young people's health and well-being. Health Behaviour in School-aged Children (HBSC) study: international report from the 2013/2014 survey.

[25] (2018). Physical actvity country fact sheets.

[26] Rotondi V, Stanca L, Tomasuolo M (2017). Connecting alone: Smartphone use, quality of social interactions and well-being. Journal of Economic Psychology.

[27] (2014). Istat Statistics. Resident municipal population by age, sex and marital status database on the Internet.

[28] Cao W, Fang Z, Hou G, Han M, Xu X, Dong J, Zheng J (2020). The psychological impact of the COVID-19 epidemic on college students in China. Psychiatry Res.

[29] Currie C, Inchley J, Molcho M, Lenzi M, Veselska Z, Wild F (2014). Health Behaviour in School-aged Children (HBSC) study protocol: background, methodology and mandatory items for the 2013/14 survey.

[30] Aarø LE, Wold B, Kannas L, Rimpelä M (1986). Health behaviour in schoolchildren. A WHO cross-national survey: A presentation of philosophy, methods and selected results of the first survey. Health Promot Int.

[31] Roberts C, Currie C, Samdal O, Currie D, Smith R, Maes L (2007). Measuring the health and health behaviours of adolescents through cross-national survey research: recent developments in the Health Behaviour in School-aged Children (HBSC) study. J Public Health.

[32] Roberts C, Freeman J, Samdal O, Schnohr CW, de Looze ME, Nic Gabhainn S, Iannotti R, Rasmussen M, International HBSC Study Group (2009). The Health Behaviour in School-aged Children (HBSC) study: methodological developments and current tensions. Int J Public Health.

[33] Kosticova M, Husarova D, Dankulincova Z (2020). Difficulties in Getting to Sleep and their Association with Emotional and Behavioural Problems in Adolescents: Does the Sleeping Duration Influence this Association?. Int J Environ Res Public Health.

[34] Gariepy G, McKinnon B, Sentenac M, Elgar FJ (2015). Validity and Reliability of a Brief Symptom Checklist to Measure Psychological Health in School-Aged Children. Child Ind Res.

[35] Bobakova D, Hamrik Z, Badura P, Sigmundova D, Nalecz H, Kalman M (2015). Test-retest reliability of selected physical activity and sedentary behaviour HBSC items in the Czech Republic, Slovakia and Poland. Int J Public Health.

[36] Liu Y, Wang M, Tynjälä J, Lv Y, Villberg J, Zhang Z, Kannas L (2010). Test-retest reliability of selected items of Health Behaviour in School-aged Children (HBSC) survey questionnaire in Beijing, China. BMC Med Res Methodol.

[37] Busschaert C, De Bourdeaudhuij I, Van Holle V, Chastin SFM, Cardon G, De Cocker K (2015). Reliability and validity of three questionnaires measuring context-specific sedentary behaviour and associated correlates in adolescents, adults and older adults. Int J Behav Nutr Phys Act.

[38] Rey-López JP, Vicente-Rodriguez G, Ortega FB, Ruiz JR, Martinez-Gómez D, De Henauw S, Manios Y, Molnar D, Polito A, Verloigne M, Castillo MJ, Sjöström M, De Bourdeaudhuij I, Moreno LA, HELENA Study Group (2010). Sedentary patterns and media availability in European adolescents: The HELENA study. Prev Med.

[39] Gopinath B, Hardy LL, Baur LA, Burlutsky G, Mitchell P (2012). Physical activity and sedentary behaviors and health-related quality of life in adolescents. Pediatrics.

[40] Bucksch J, Kopcakova J, Inchley J, Troped PJ, Sudeck G, Sigmundova D, Nalecz H, Borraccino A, Salonna F, Dankulincova Veselska Z, Hamrik Z (2019). Associations between perceived social and physical environmental variables and physical activity and screen time among adolescents in four European countries. Int J Public Health.

[41] Buja A, Gallimberti L, Chindamo S, Lion C, Terraneo A, Rivera M, Marini E, Gomez-Perez LJ, Scafato E, Baldo V (2018). Problematic social networking site usage and substance use by young adolescents. BMC Pediatr.

[42] Li J, Lau JTF, Mo PKH, Su X, Wu AMS, Tang J, Qin Z (2016). Validation of the Social Networking Activity Intensity Scale among Junior Middle School Students in China. PLoS One.

[43] Cole TJ, Bellizzi MC, Flegal KM, Dietz WH (2000). Establishing a standard definition for child overweight and obesity worldwide: international survey. BMJ.

[44] Aasvee K, Rasmussen M, Kelly C, Kurvinen E, Giacchi MV, Ahluwalia N (2015). Validity of self-reported height and weight for estimating prevalence of overweight among Estonian adolescents: the Health Behaviour in School-aged Children study. BMC Res Notes.

[45] Elgar FJ, Roberts C, Tudor-Smith C, Moore L (2005). Validity of self-reported height and weight and predictors of bias in adolescents. J Adolesc Health.

[46] Lee J, Choi MJ, Thornberg R, Hong JS (2020). Exploring Sex Differences in the Association between Bullying Involvement and Alcohol and Marijuana Use among U.S. Adolescents in 6th to 10th Grade. Subst Use Misuse.

[47] Horn-Hofmann Claudia, Trost Zina, Hublet Anne, Mrug Sylvie, Van Damme Joris, Vervoort Tine (2018). The Relationship Between Pain Severity and Alcohol Use Among School-Aged Children and Adolescents: The Moderating Role of Drinking Motives. Pain Medicine.

[48] Sigmundová D, Sigmund E, Badura P, Vokáčová J, Trhlíková L, Bucksch J (2016). Weekday-weekend patterns of physical activity and screen time in parents and their pre-schoolers. BMC Public Health.

[49] Alsaggaf MA, Wali SO, Merdad RA, Merdad LA (2016). Sleep quantity, quality, and insomnia symptoms of medical students during clinical years. Relationship with stress and academic performance. Saudi Med J.

[50] Ramamoorthy S, Kamaldeen D, Ravichandran L, Sundaramahalingam M (2019). Effect of stress on sleep hygiene among school going adolescents in Chennai. J Family Med Prim Care.

[51] American Academy of Pediatrics. Committee on Public Education (2001). American Academy of Pediatrics: Children, adolescents, and television. Pediatrics.

[52] Irish LA, Kline CE, Rothenberger SD, Krafty RT, Buysse DJ, Kravitz HM, Bromberger JT, Zheng H, Hall MH (2014). A 24-hour approach to the study of health behaviors: temporal relationships between waking health behaviors and sleep. Ann Behav Med.

[53] Kelly Y, Zilanawala A, Booker C, Sacker A (2018). Social Media Use and Adolescent Mental Health: Findings From the UK Millennium Cohort Study. EClinicalMedicine.

[54] Carter B, Rees P, Hale L, Bhattacharjee D, Paradkar MS (2016). Association Between Portable Screen-Based Media Device Access or Use and Sleep Outcomes: A Systematic Review and Meta-analysis. JAMA Pediatr.

[55] Shochat T, Flint-Bretler O, Tzischinsky O (2010). Sleep patterns, electronic media exposure and daytime sleep-related behaviours among Israeli adolescents. Acta Paediatr.

[56] Lemola S, Perkinson-Gloor N, Brand S, Dewald-Kaufmann JF, Grob A (2015). Adolescents' electronic media use at night, sleep disturbance, and depressive symptoms in the smartphone age. J Youth Adolesc.

[57] Dorofaeff TF, Denny S (2006). Sleep and adolescence. Do New Zealand teenagers get enough?. J Paediatr Child Health.

[58] Punamäki R, Wallenius M, Nygård C, Saarni L, Rimpelä A (2007). Use of information and communication technology (ICT) and perceived health in adolescence: the role of sleeping habits and waking-time tiredness. J Adolesc.

[59] Amra B, Shahsavari A, Shayan-Moghadam R, Mirheli O, Moradi-Khaniabadi B, Bazukar M, Yadollahi-Farsani A, Kelishadi R (2017). The association of sleep and late-night cell phone use among adolescents. J Pediatr (Rio J).

[60] Munezawa T, Kaneita Y, Osaki Y, Kanda H, Minowa M, Suzuki K, Higuchi S, Mori J, Yamamoto R, Ohida T (2011). The association between use of mobile phones after lights out and sleep disturbances among Japanese adolescents: a nationwide cross-sectional survey. Sleep.

[61] Troxel WM, Hunter G, Scharf D (2015). Say "GDNT": Frequency of Adolescent Texting at Night. Sleep Health.

[62] (2019). Dipendenza da Internet, arriva la legge per curarla. Il Messaggero.

[63] Woods HC, Scott H (2016). #Sleepyteens: Social media use in adolescence is associated with poor sleep quality, anxiety, depression and low self-esteem. J Adolesc.

[64] Wolniczak I, Cáceres-DelAguila JA, Palma-Ardiles G, Arroyo KJ, Solís-Visscher R, Paredes-Yauri S, Mego-Aquije K, Bernabe-Ortiz A (2013). Association between Facebook dependence and poor sleep quality: a study in a sample of undergraduate students in Peru. PLoS One.

[65] Levenson JC, Shensa A, Sidani JE, Colditz JB, Primack BA (2016). The association between social media use and sleep disturbance among young adults. Prev Med.

[66] Harbard E, Allen NB, Trinder J, Bei B (2016). What's Keeping Teenagers Up? Prebedtime Behaviors and Actigraphy-Assessed Sleep Over School and Vacation. J Adolesc Health.

[67] Scott H, Woods HC (2018). Fear of missing out and sleep: Cognitive behavioural factors in adolescents' nighttime social media use. J Adolesc.

[68] de Bérail P, Guillon M, Bungener C (2019). The relations between YouTube addiction, social anxiety and parasocial relationships with YouTubers: A moderated-mediation model based on a cognitive-behavioral framework. Computers in Human Behavior.

[69] Erwin AM, Bashore L (2017). Subjective Sleep Measures in Children: Self-Report. Front Pediatr.

[70] Wolfson AR, Carskadon MA, Acebo C, Seifer R, Fallone G, Labyak SE, Martin JL (2003). Evidence for the validity of a sleep habits survey for adolescents. Sleep.

[71] Chambers MJ (1994). Actigraphy and insomnia: a closer look. Part 1. Sleep.

[72] Zinkhan M, Berger K, Hense S, Nagel M, Obst A, Koch B, Penzel T, Fietze I, Ahrens W, Young P, Happe S, Kantelhardt JW, Kluttig A, Schmidt-Pokrzywniak A, Pillmann F, Stang A (2014). Agreement of different methods for assessing sleep characteristics: a comparison of two actigraphs, wrist and hip placement, and self-report with polysomnography. Sleep Med.

[73] Wetter DW, Young TB (1994). The relation between cigarette smoking and sleep disturbance. Prev Med.

[74] Pallesen S, Hetland J, Sivertsen B, Samdal O, Torsheim T, Nordhus IH (2008). Time trends in sleep-onset difficulties among Norwegian adolescents: 1983--2005. Scand J Public Health.

[75] Mosconi P, Radrezza S, Lettieri E, Santoro E (2019). Use of Health Apps and Wearable Devices: Survey Among Italian Associations for Patient Advocacy. JMIR Mhealth Uhealth.

[76] Espinoza G, Juvonen J (2011). The pervasiveness, connectedness, and intrusiveness of social network site use among young adolescents. Cyberpsychol Behav Soc Netw.

[77] Un bambino su quattro soffre di disturbi del sonno. Le 10 regole d'oro per far dormire i piccoli, i più grandi (e anche mamma e papà). Società Italiana di Pediatria.

[78] (2018). Life in ‘likes’: Children’s Commissioner report into social media use among 8-12 year olds.

