# Supplementary material for: The Role of Technology and Social Media Use in Sleep-Onset Difficulties Among Italian Adolescents: Cross-sectional Study
Source: J Med Internet Res. 2021 Jan 21;23(1):e20319. doi: 10.2196/20319 (PMC7862002; doi:10.2196/20319)
Supplement: Multimedia Appendix 1 [file jmir_v23i1e20319_app1.docx]

# **Supplementary Tables**

| **Supplementary Table S1.** Distribution of 3172 adolescents with sleep-onset difficulties overall and by technology use during school days and weekends. Corresponding odds ratios° (OR) and 95% confidence intervals (CI) for the full sample and sex-specific samples. Lombardy, 2014. | | | | | | | | | |
| --- | --- | --- | --- | --- | --- | --- | --- | --- | --- |
|  | **Total** | | | **Males** | | | **Females** | | |
|  | **N*** | **Difficulty falling asleep** | | **N** | **Difficulty falling asleep** | | **N** | **Difficulty falling asleep** | |
|  |  | **%** | **OR (95% CI)** |  | **%** | **OR (95% CI)** |  | **%** | **OR (95% CI)** |
| **Total screen time-weekday** |  |  |  |  |  |  |  |  |  |
| 1^st^ tertile (<2 hours/day) | 1217 | 29.6 | 1.00^ | 592 | 26.0 | 1.00^ | 625 | 33.1 | 1.00^ |
| 2^nd^ tertile (2-3.9 hours/day) | 853 | 34.5 | 1.21 (0.98-1.48) | 463 | 27.4 | 0.99 (0.74-1.34) | 390 | 42.8 | 1.42 (1.06-1.90) |
| 3^rd^ tertile (≥4 hours/day) | 1102 | 39.3 | 1.41 (1.16-1.72) | 582 | 35.3 | 1.50 (1.14-1.98) | 520 | 43.7 | 1.30 (0.98-1.72) |
| p for trend |  |  | .001 |  |  | .004 |  |  | .072 |
| **Total screen time-weekend** |  |  |  |  |  |  |  |  |  |
| 1^st^ tertile (<2.5 hours/day) | 1160 | 29.9 | 1.00^ | 561 | 27.4 | 1.00^ | 599 | 32.2 | 1.00^ |
| 2^nd^ tertile (2.5-4.9 hours/day) | 872 | 32.7 | 1.19 (0.96-1.46) | 475 | 23.9 | 0.84 (0.62-1.14) | 397 | 43.2 | 1.61 (1.20-2.16) |
| 3^rd^ tertile (≥5 hours/day) | 1140 | 40.0 | 1.51 (1.24-1.83) | 601 | 36.5 | 1.48 (1.13-1.94) | 539 | 44.0 | 1.47 (1.11-1.94) |
| p for trend |  |  | <.001 |  |  | .004 |  |  | .007 |
| *The sum does not add up to the total because of some missing values and exclusion of age 11 for social media measures. °ORs were estimated using unconditional multiple logistic regression models after adjustment for age and sex of the child, mothers and fathers’ highest level of education, tobacco and alcohol use among adolescents and BMI.  ^ Reference category | | | | | | | | | |

| **Supplementary Table S2.** Odds ratios° (OR) and 95% confidence intervals (CI) of sleep onset difficulties when including indicators for school of the adolescent in addition to the baseline model. Lombardy, 2014 | |
| --- | --- |
|  | **OR (95% CI)** |
| **Electronic device use for general purpose** |  |
| 1^st^ tertile (<0.9 hours/day) | 1.00^ |
| 2^nd^ tertile (0.9-2.1 hours/day) | 1.14 (0.91-1.42) |
| 3^rd^ tertile (≥2.2 hours/day) | 1.44 (1.16-1.81) |
| p for trend | .001 |
| **Electronic device use for playing games** |  |
| 1^st^ tertile (<0.8 hours/day) | 1.00^ |
| 2^nd^ tertile (0.8-1.7 hours/day) | 1.15 (0.92-1.44) |
| 3^rd^ tertile (≥1.8 hours/day) | 1.28 (1.03-1.58) |
| p for trend | .024 |
| **Use of social networking sites** |  |
| Never/Rarely^ | 1.00^ |
| Often | 1.16 (0.89-1.52) |
| Always | 1.47 (1.11-1.95) |
| p for trend | .007 |
| **Use of YouTube** |  |
| Never/Rarely^ | 1.00^ |
| Often | 1.63 (1.21-2.21) |
| Always | 2.15 (1.57-2.95) |
| p for trend | <.001 |
| °ORs were estimated using multiple logistic regression models after adjustment for age and sex of the child mothers and fathers highest level of education, tobacco and alcohol use among adolescents, and BMI and school indicators of the participant.  ^Reference category | |

| **Supplementary Table S3.** Distribution of 3172 adolescents with sleep-onset difficulties overall and by technology use using alternative definitions of exposure and outcome variables. Corresponding odds ratios (OR) and 95% confidence intervals (CI) for the full sample and sex-specific samples. Lombardy, 2014. | | | | | | | | | |
| --- | --- | --- | --- | --- | --- | --- | --- | --- | --- |
|  | **Total** | | |  | **Males** | |  | **Females** | |
|  | **N*** | **Difficulty falling asleep** | | **N** | **Difficulty falling asleep** | | **N** | **Difficulty falling asleep** | |
|  |  | **%** | **OR (95% CI)** |  | **%** | **OR (95% CI)** |  | **%** | **OR (95% CI)** |
| **Total time spend on electronic devices** |  |  |  |  |  |  |  |  |  |
| Less than or equal to 2 hours/day | 1251 | 17.9 | 1.00^ | 615 | 14.1 | 1.00^ | 636 | 21.5 | 1.00^ |
| Exceeding 2 hours per day | 1921 | 24.7 | 1.52 (1.24-1.87) | 1022 | 19.6 | 1.46 (1.08-1.98) | 899 | 30.5 | 1.55 (1.18-2.04) |
|  |  |  |  |  |  |  |  |  |  |
| **Use of social networking sites** |  |  |  |  |  |  |  |  |  |
| Never/Rarely | 701 | 19.8 | 1.00^ | 397 | 15.1 | 1.00^ | 304 | 26.0 | 1.00^ |
| Often | 650 | 22.3 | 1.10 (0.83-1.47) | 345 | 18.6 | 1.29 (0.85-1.98) | 305 | 26.6 | 1.00 (0.68-1.48) |
| Always | 599 | 28.7 | 1.43 (1.07-1.90) | 255 | 20.0 | 1.57 (1.00-2.44) | 344 | 35.2 | 1.38 (0.94-2.02) |
| p for trend |  |  | .015 |  |  | .048 |  |  | .097 |
|  |  |  |  |  |  |  |  |  |  |
| **Use of YouTube** |  |  |  |  |  |  |  |  |  |
| Never/Rarely | 412 | 17.7 | 1.00^ | 209 | 12.4 | 1.00^ | 203 | 23.2 | 1.00^ |
| Often | 854 | 22.1 | 1.32 (0.96-1.84) | 434 | 16.6 | 1.25 (0.75-2.09) | 420 | 27.9 | 1.39 (0.91-2.13) |
| Always | 683 | 28.4 | 1.86 (1.34-2.59) | 354 | 21.8 | 1.90 (1.14-3.17) | 329 | 35.6 | 1.79 (1.15-2.78) |
| p for trend |  |  | <.001 |  |  | .014 |  |  | .010 |
| *The sum does not add up to the total because of some missing values and exclusion of age 11 for social media measures. °ORs were estimated using unconditional multiple logistic regression models after adjustment for age and sex of the child, mothers and fathers’ highest level of education, tobacco and alcohol use among adolescents and BMI.  ^Reference category | | | | | | | | | |

| **Supplementary Table S4.** Odds ratios° (OR) and 95% confidence intervals (CI) of sleep onset difficulties controlling for perceived economic status of the family in addition to the baseline model. Lombardy, 2014 | |
| --- | --- |
|  | **OR (95% CI)** |
| **Electronic device use for general purpose** |  |
| 1^st^ tertile (<0.9 hours/day) | 1.00^ |
| 2^nd^ tertile (0.9-2.1 hours/day) | 1.15 (0.94-1.42) |
| 3^rd^ tertile (≥2.2 hours/day) | 1.49 (1.21-1.84) |
| p for trend | <.001 |
| **Electronic device use for playing games** |  |
| 1^st^ tertile (<0.8 hours/day) | 1.00^ |
| 2^nd^ tertile (0.8-1.7 hours/day) | 1.21 (0.98-1.49) |
| 3^rd^ tertile (≥1.8 hours/day) | 1.33 (1.09-1.62) |
| p for trend | .005 |
| **Use of social networking sites** |  |
| Never/Rarely^ | 1.00^ |
| Often | 1.17 (0.91-1.49) |
| Always | 1.43 (1.11-1.84) |
| p for trend | .006 |
| **Use of YouTube** |  |
| Never/Rarely^ | 1.00^ |
| Often | 1.57 (1.19-2.08) |
| Always | 2.02 (1.51-2.70) |
| p for trend | <.001 |
| °ORs were estimated using multiple logistic regression models after adjustment for age and sex of the child, mothers and fathers’ highest level of education, tobacco and alcohol use among adolescents, and BMI and perceived economic status of the family.  ^Reference category | |
